# Acoustic radiation pressure for nonreciprocal transmission and switch effects

**DOI:** 10.1038/s41467-019-11305-7

**Published:** 2019-07-23

**Authors:** Thibaut Devaux, Alejandro Cebrecos, Olivier Richoux, Vincent Pagneux, Vincent Tournat

**Affiliations:** Laboratoire d’Acoustique de l’Université du Mans, LAUM UMR 6613, CNRS, Le Mans Université, Av. O. Messiaen, 72085 Le Mans, France

**Keywords:** Mechanical engineering, Acoustics

## Abstract

Systems capable of breaking wave transmission reciprocity have recently led to tremendous developments in wave physics. We report herein on a concept that enables one-way transmission of ultrasounds, an acoustic diode, by relying on the radiation pressure effect. This effect makes it possible to reconfigure a multilayer system by significantly deforming a water-air interface. Such a reconfiguration is then used to achieve an efficient acoustic transmission in a specified direction of propagation but not in the opposite, hence resulting in a highly nonreciprocal transmission. The corresponding concept is experimentally demonstrated using an aluminum-water-air-aluminum multilayer system, providing the means to overcome key limitations of current nonreciprocal acoustic devices. We also demonstrate that this diode functionality can even be extended to the design and operations of an acoustic switch, thus paving the way for new wave control possibilities, such as those based on acoustic transistors, phonon computing and amplitude-dependent filters.

## Abstract (web summary)

Here, the authors experimentally report one-way transmission of ultrasonic waves by relying on the acoustic radiation pressure effect. This effect makes it possible to reconfigure a multilayer system by significantly deforming the water-air interface, achieving efficient nonreciprocal acoustic transmission.

## Introduction

In the field of wave physics and its applications in areas such as imaging, major efforts have recently targeted nonreciprocal transmission effects derived from architected devices or materials. This widespread interest, most certainly fueled by the prevalence of electrical diodes in today’s human activities, is apparent for instance in optics^1–8^ and in thermal physics as thermal diodes and transistors^9–12^, with applications aimed, for example, at the key components of optical computers and devices for thermal energy control and harvesting. In the field of acoustics, nonreciprocal structures with strong asymmetry in their transmission properties have been proposed^13,14^ and cited as candidates for promoting an array of innovations in ultrasonic nondestructive testing, vibrational protection, information processing, medical imaging, or even in energy harvesting^15–20^.

The most commonly encountered means for breaking the reciprocity of a wave system is to induce nonlinearity into the propagation process or to have the wave interact with a localized nonlinear element^13–19,21,22^. Asymmetric devices based on such wave nonlinearity typically feature a conversion stage, as assumed by a highly nonlinear medium or element, plus a selection stage via filtering by, for example, phononic or photonic crystals. These systems often exhibit a strong contrast between energies transmitted in opposite propagation directions, as denoted by the asymmetry ratio^15–18^. As previously noted, however, the nonlinear mechanisms in play induce a frequency conversion of the initial wave during the transmission process, that is, towards the second harmonic wave^15,16,23^ or the self-demodulated component^18^, thus leading to a distortion of both the signal and the information being carried, as well as to an energy transmission coefficient of much <1. Strategies to overcome this limitation are still to be found and tested^24^. Moreover, such devices based on nonlinear frequency conversion effects normally require a precise design and only operate at specific frequencies, even in spite of the operational bandwidth improvements proposed in ref. ^18^.

Several other strategies for breaking a system reciprocity have recently been explored, such as those relying on an external bias imposed on the acoustic wave field, for example, a circulating air flow^25^, those introducing active electro-acoustic elements^26–28^, thermo-acoustic elements^29^, or those using an appropriate time modulation of the propagation medium properties^30,31^. These systems offer the advantages of: accommodating a relatively high asymmetry ratio between two given ports, efficiently transmitting the same frequency as the incident wave, and for some of them operating over a broad frequency range. Although extremely promising, all such systems still require the input of external energy, since they are not fully activated by the waves themselves and thus cannot be considered as passive. Moreover, one-way, topologically protected edge waves, in an analogy with the quantum Hall effect or the quantum spin Hall effect^32^, have also attracted considerable attention over the past several years. These waves offer extensive possibilities, yet are also currently restricted by both the complexity of the material design, that is, rotating fluids or gyroscopes, and their operations, that is, coupling of an external wave mode with the coupled one-way right spin–orbit mode, thus limiting their applicability at the present time as acoustic diodes.

In this article, we propose a heretofore unexplored strategy to break the transmission symmetry of a system. We make use of the quasi-static deformation of a fluid layer within a multilayer propagation medium originating from the acoustic radiation pressure (RP) effect. This deformation, which in our case can only occur for one incidence direction of the ultrasonic waves, leads to a reversible reconfiguration of the multilayer system. The several states accessible by this system reconfiguration reveal drastic differences in the succession of impedance jumps at each interface, thus capable of drastically altering the transmission properties. The performance of a prototype is demonstrated experimentally herein as well, in displaying a typical asymmetric transmission ratio along with a high transmission of acoustic energy in the forward direction, yet without any significant signal distortion. Moreover, the proposed concept relies on a relatively simple system design and can operate over a wide range of frequencies; however, still needing the gravity as an external bias. In light of its ability to overcome some of the major limitations of previously reported processes, this concept will be extended in the last section in order to produce an acoustic switch.

## Results

### Principle of the nonreciprocal transmission device

**Fig. 1:**
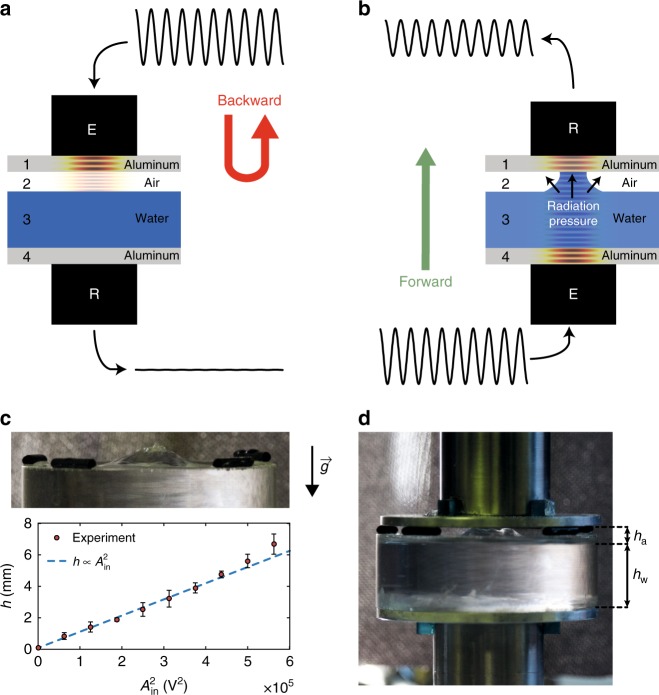
Schematic principle of the concept and a picture of the prototype. **a**, **b** Schematic diagrams of the forward (emitter E at the bottom, receiver R at the top) and backward (emitter E at the top, receiver R at the bottom) transmission configurations, respectively. **c** Radiation pressure effect on the water surface quasi-static deformation. Picture of the water bump, and experimental height as a function of the input electrical amplitude squared (error bars correspond to the standard deviation of 50 data points collected over 10 s for each amplitude); **d** picture of the tested device when forward transmission is active

The designed device is a multilayer system composed of four successive layers. On both exterior sides, 3-mm-thick aluminum layers closing the system are placed in contact with the piezoelectric transducers, with the intention of generating and detecting the ultrasonic waves to be transmitted through the device, as shown in Fig. 1a, b, d. The two inner layers are composed, respectively, of water and air, with naturally strong acoustic impedance jumps at each interface *ρ*_a_*c*_a_/*ρ*_w_*c*_w_ ≈ 2 × 10^−3^ and *ρ*_a_*c*_a_/*ρ*_Al_*c*_Al_ ≈ 2 × 10^−5^, where *ρ*_i_ are the densities and *c*_i_ the acoustic wave speeds in air, water, and aluminum layers (with indices a, w, and Al, respectively). The transmitted ultrasonic amplitude through this system can be measured in both directions of operation, denoted as either “backward” or “forward,” by inverting the roles of the identical piezoelectric transducers in order to characterize the transmission asymmetry.

In the “backward” direction, the piezo-emitter first excites layer 1 (solid aluminum) from the top (see Fig. 1a). The mechanical impedance of layer 2 (air) is at least four orders of magnitude less than layer 1, thus ensuring a nearly complete reflection of the wave energy at this interface. The next interface between layers 2 and 3 (water) constitutes another strong impedance jump; lastly, the transmission of a signal through the device is barely observable, as will be shown further below. In the “forward” direction (Fig. 1b), layer 4 (aluminum) is excited first, and a significant portion of the ultrasonic energy is also transmitted to layer 3 (water). When the excitation amplitude is increased, a quasi-static deformation of the air/water interface is generated due to the acoustic RP^33–36^. Like in the field of optics, the nonlinear acoustic effect of RP has been studied in the contexts of lab-on-chip/microfluidics, medical imaging, surface tension characterization, acoustic levitation, fountain, or even tweezers^37–39^, in addition to fundamental research and the seminal results found by Rayleigh^40^, Brillouin, or Langevin in the early twentieth century. Besides these advances, the RP effect demonstrates its robustness for the reconfiguration in real time of bubbly propagation media and their dispersion properties^41,42^, with wave control applications. The RP generated at a water–air interface by an axisymmetric ultrasonic beam incoming orthogonally from water has been quantified by the Langevin radiation stress tensor^35^ as:

1 $${\Pi}(r) = 2\langle p_{\mathrm{i}}^2(r,t)\rangle _t/\rho _{\mathrm{w}} c_{\mathrm{w}} ^2,$$

where *r* is the radial space variable, $p_{\mathrm{i}}^2(r,t)$ is the square of the incident acoustic pressure on the interface, *ρ*_w_ the density, and *c*_w_ the wave speed in water. Moreover, 〈…〉_*t*_ denotes temporal averaging, that is, in correspondence with the quasi-static or direct current contribution. Π is of maximum value at the beam center *r* = 0 for a typical beam profile; RP then increases locally the average water height (see Supplementary Note 2), causing a bump around *r* = 0 that is capable of reaching a few millimeter in height, ultimately filling the air gap for sufficiently large *p*_i_ and creating a water–aluminum (layer 3–layer 1) contact. As expected from theory and observed experimentally in Fig. 1c, the bump height at its center is expressed as:

2 $$h(r = 0) \propto \langle p_{\mathrm{i}}^2(0,t)\rangle _t \propto A_{{\mathrm{in}}}^2,$$

where *A*_in_ is the electrical excitation amplitude. The water–air interface deformation is found to be stable in the configuration shown in Fig. 1c up to *h*(*r* = 0) ≈ 5 mm. In the device, if the air-gap thickness *h*_a_ < 5 mm, then the successive impedance jumps at both the water–air and air–aluminum interfaces can be reduced to the relatively much smaller impedance jump found at the newly formed water–aluminum interface. Note that the latter and the associated contact surface are stable in time. By modifying the successive impedance mismatches over several orders of magnitude, the multilayer system reconfiguration due to water–air interface deformation induced by RP thus drastically alters transmission properties. Note that this effect can be achieved in the forward, but not in the backward, direction, hence leading to a strongly nonreciprocal and amplitude-dependent transmission, analogous to a diode characteristic. Once this contact has been achieved in the “forward” direction, however, another wave could propagate in the backward direction, which is a feature not replicated in an isolator. Noticeably, the layer deformation by RP and the subsequent reconfiguration of the multilayer system is in direct analogy with a p-n junction electrical diode, the air layer playing here a role similar to the depletion zone between the p- and n-doped regions. We finally note that passive linear time-invariant systems such as those reported in refs. ^43–45^ are reciprocal and do not qualify as “acoustic diodes” or “acoustic nonreciprocal transmission” devices, as noted in ref. ^13^. They actually do not violate the Rayleigh reciprocity theorem^14^, while our system, due to the nonlinear RP process, breaks the Rayleigh reciprocity theorem and is consequently nonreciprocal.

### Direction and amplitude-dependent transmission

A device utilizing this concept has been built and tested (see Fig. 1d), with an air layer thickness *h*_a_ capable of being adjusted by means of changing the height of the top aluminum disc in contact with the top transducer. The water layer consists of a water-filled PMMA tube closed at the bottom by an aluminum disc, below which the bottom transducer is glued. The two identical piezoelectric transducers are thus placed on both aluminum ends of the device and, depending on the propagation direction, chosen to respectively excite or detect the ultrasonic waves. The wide-band ultrasonic emitter can be excited with a sine signal over a range of frequencies centered at 2.25 MHz, that is, its central frequency, extending to peak-to-peak electrical amplitudes of 750 V. The limit of 750 V, providing estimated acoustic pressures of several hundreds of kPa, is chosen both for not damaging the emitter and for avoiding important cavitation effects in water, the associated acoustic power being close to the cavitation threshold (see Supplementary Note 2).

**Fig. 2:**
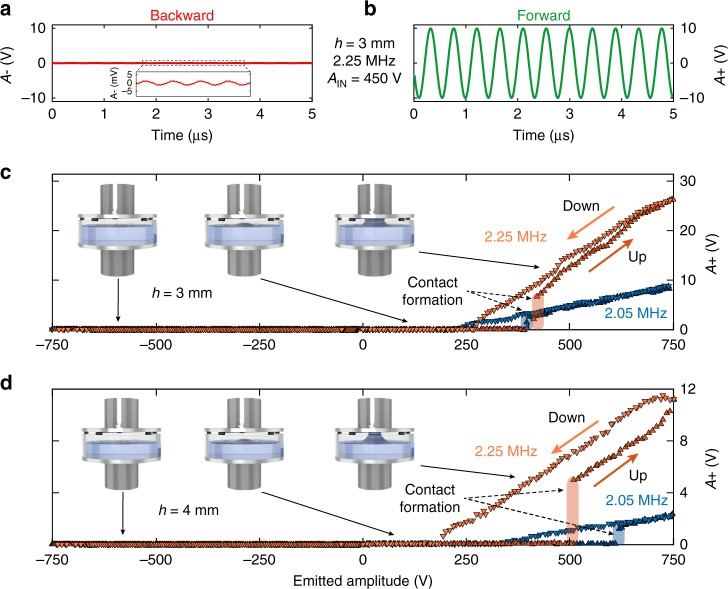
Temporal signals and amplitude dependence. Transmitted amplitude *A*+ for an excitation frequency of 2.25 MHz and input amplitude *V*_in_ = 450 V, with an air layer thickness *h*_a_ = 3 mm in: **a** backward and **b** forward directions. The inset in **a** is a close-up of the transmitted signal in the backward direction. **c** Transmitted amplitude in both the backward (*A*−) and forward (*A*+) propagation directions as a function of the emitted amplitude *V*_in_ along a cycle of increasing and decreasing amplitudes, for two different excitation frequencies (*f*_1_ = 2.05 MHz and *f*_2_ = 2.25 MHz) and two different air layer thicknesses: *h*_a_ = 3 mm in **c** and *h*_a_ = 4 mm in **d**. The diagrams in **c**, **d** illustrate the multilayer configuration at the indicated emitted amplitudes. The negative (positive) emitted amplitude horizontal scale corresponds to the peak-to-peak amplitude of the signal emitted in the backward (forward) direction, respectively

As could be expected from the large impedance mismatches between layers in the “backward” configuration (Fig. 2a), the signal amplitude transmitted through the device, *A*−, is weak, approx. *A*−≈1 mV. Conversely, in the “forward” direction, the transmitted signal amplitude in the typical example is *A*+ ≈10 V. In a reciprocal system, for example, a classical multilayer system, the signals in the backward (Fig. 2a) and forward (Fig. 2b) directions would be of the same level, while in the present case they display a four orders-of-magnitude difference. In addition, the transmitted signal has the same frequency as the emitted signal, as opposed to the nonlinear nonreciprocal systems previously proposed in acoustics, all of which imply a frequency change, either toward higher harmonics^15^ or lower frequencies^17–19^. The fact that the transmitted signals appear to be non-distorted in Fig. 2b is attributed to both the weak second harmonic generation efficiency and the lack of sensitivity in the piezo-detector above 3 MHz.

Let us now turn our attention to analyzing the amplitude-dependent transmission in the forward and backward transmission directions. To proceed, we performed up-and-down amplitude sweeps in both transmission directions, from 0 to 750 V and back to 0 V in a 25-s time interval. The detected amplitude as a function of excitation amplitude is shown in Fig. 2c, d for two ultrasonic frequencies and two initial air-gap thicknesses. In all cases, the detected signal magnitude in the backward direction lies close to zero for any excitation amplitude (shown as a negative signal magnitude in order to denote the backward transmission direction). In contrast, the detected signal magnitude in the forward direction is initially near zero and then suddenly increases, with a threshold effect, when the excitation amplitude increases. This threshold effect is due to the formation of a water bump via the RP, which starts to touch the top aluminum plate. The non-transmitting four-layer, aluminum–water–air–aluminum system is thus reconfigured at this threshold into a transmitting three-layer, aluminum–water–aluminum system. The thicker the air gap, the higher the excitation amplitude where the threshold occurs (respectively at around 400 V for *h*_a_ = 3 mm and over 500 V for *h*_a_ = 4 mm). Moreover, this threshold depends on the exact excitation frequency, mainly as a result of the transducer frequency response and the remnant effect of the Fabry–Perot water layer resonances, as will be analyzed and discussed below.

Another significant effect is the hysteretic characteristic of the detected amplitude *A*+ as a function of the excitation amplitude sweep. The primary contribution to the observed hysteresis is the water capillary force, which tends to maintain the water bump–aluminum contact as the excitation amplitude slowly decreases, while it plays no role in the increasing amplitude stage, at least whenever the water–aluminum contact is not yet active. The threshold is therefore distinct in the increasing excitation amplitude stage while the detected amplitude does not exhibit any clear threshold during the decreasing excitation amplitude stage. The remnant hysteresis, in the form of a small difference in detected amplitude *A*+ in the increasing and decreasing excitation amplitude stages (which is also visible in the case without air gap exposed in Supplementary Fig. 3 of the Supplementary Note 2), can be attributed to the system temperature change (water and transducer) due to high power excitation and acoustic energy dissipation. Note that the transmitted amplitude in the three-layer case (no initial air gap) is observed up to *A*+ ≈30 V, as will be described subsequently. This observation would be expected as a maximum transmitted amplitude in the forward direction of the asymmetric device with RP, and is limited by the transduction efficiency of both emitter and receiver. Note that performing a meaningful measure of the transmission coefficient by this device is a difficult task since the incoming and outgoing waves cannot be isolated due to multiple reflections on the transducers. Prediction of the transmission coefficient is also made difficult because of the deviation from the one-dimensional geometry (ultrasonic beam, water layer deformation), and losses. Although there are aluminum–water impedance mismatches in the multilayer, internal interferences can lead to transmission coefficients close to 1 for some particular frequencies, and *A*+ ≈30 V found for the three-layer case corresponds most probably to a transmission of the same order of magnitude than 1. The transmitted amplitude up to *A*+ ≈26 V is observed in Fig. 2c, thus demonstrating an exceptional transmission efficiency for our system compared to most existing nonlinear asymmetric devices, for example, those leading to a frequency conversion^15,17–19^.

**Fig. 3:**
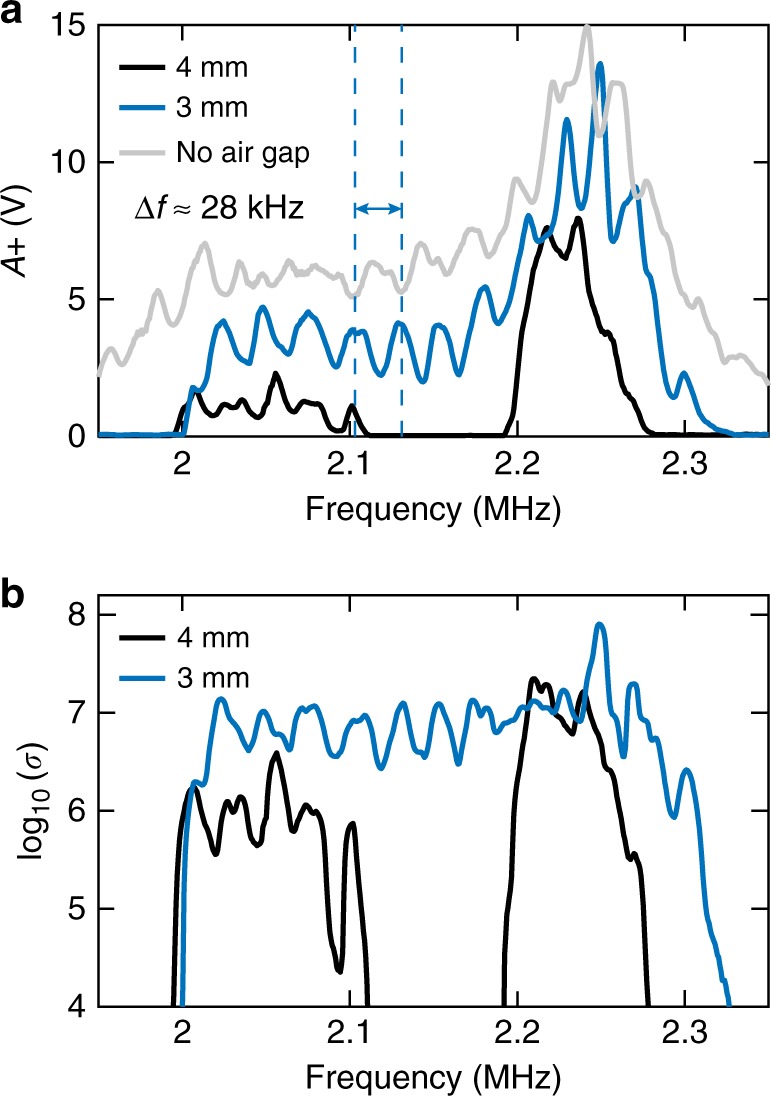
Experimental frequency response of the diode effect: **a** Transmitted amplitude in the forward direction (*A*+) vs. excitation frequency for the diode system with two different air-gap thicknesses, as well as for the reference case without an air gap. The excitation amplitude is *V*_in_ = 500 V. **b** Transmission asymmetry ratio *σ* = *A*+^2^/*A*−^2^ vs. frequency for two distinct air-gap thicknesses

The results presented for two excitation frequencies exhibit clear differences, such as in the threshold amplitude or the detected signal magnitude. These differences can be explained both by transducer sensitivity, which is frequency dependent, and by the existence of successive resonances in the water layer. In Fig. 3a, the transmitted amplitude is plotted as a function of frequency for the same excitation amplitude *V*_in_ = 500 V and for two air-gap thickness values, as well as for the reference case without any air gap (i.e., a three-layer aluminum–water–aluminum system). The oscillations repeated every ≈28 kHz correspond to the successive Fabry–Perot resonances of the *h*_w_ + *h*_a_ = 26-mm-thick water layer. Note that *h*_w_ could be increased or decreased, which would change the distance between these resonances. However, *h*_w_ cannot be made arbitrarily small since enough water is needed to form the water bump, and typically *h*_w_ >3–4 mm is needed in our configuration. Due to ultrasonic damping effects and deviations from the one-dimensional characteristic of the deformed medium and the acoustic field, these resonances are highly reduced when compared to those expected for a perfect one-dimensional multilayer system. Also, they do not play a crucial role herein since asymmetric transmission occurs out of these resonances too. In addition to these resonances, the main trend in the case of zero air gap corresponds well to the transducer frequency response, in showing a main peak at 2.25 MHz and a secondary peak of relatively lower sensitivity at 2.05 MHz. As the air-gap thickness *h*_a_ increases, the lowest-amplitude frequency regions, for example, below 2 MHz or between 2.1 and 2.2 MHz for *h*_a_ = 4 mm, actually lie below the threshold and no transmission is observed. When transmission still occurs, it is observed to be lower for *h*_a_ = 4 mm than for *h*_a_ = 3 mm, mainly due to the lateral size of the water neck filling the air-gap layer. Let us also note that the oscillations in *A*+ are slightly shifted in frequency depending on *h*_a_ since the effective water layer thicknesses, and in turn the associated resonance frequencies, depend on *h*_a_.

One important parameter already used to characterize the transmission asymmetry of a device, in addition to transmissivity in the forward direction, is the asymmetry ratio, defined here as the ratio of energy transmissions in the forward and backward directions. This asymmetry ratio can thus be expressed as *σ* = *A*+^2^/*A*−^2^, and it has been plotted as a function of frequency in Fig. 3b. A large ratio value of *σ* ≈ 10^7^ is observed over a broad frequency range, that is, 2–2.3 MHz, for both of the air gaps presented, with a maximum close to *σ* ≈ 10^8^. Due to the lower transmitted amplitude *A*+ for *h*_a_ = 4 mm, *σ* is on average smaller in this case because *A*− is similarly small for every *h*_a_ and frequency. The results described here have also been observed with other tested couples of piezoelectric transducers centered at 100 kHz and 1 MHz (see Supplementary Figs. 1 and 2 of the Supplementary Note 1), thus demonstrating the concept universality and its applicability to various frequency ranges.

### Acoustic switch effect

**Fig. 4:**
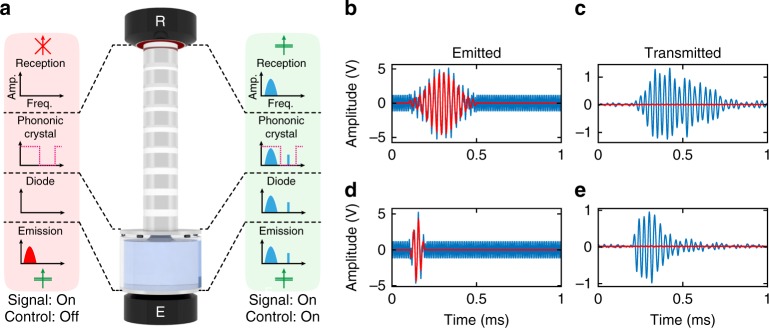
Experimental demonstration of the underlying switch principle: **a** diagrams of the switch prototype; a phononic crystal (PC) has been added on top of the acoustic diode. The switch working principle is schematically described by the effect of activating a control wave on the transmitted signal wave. **b**, **d** emitted electrical waveforms (before a +60 dB amplification by the power amplifier) for a harmonic control signal of frequency kHz and a signal wave in the form of a Gaussian pulse with a carrier frequency *f*_0_ = 35 kHz and a spectral full width at half maximum of 5 kHz (**b**) and 20 kHz (**d**). **c**, **e** output signals transmitted by the switch device. The continuous red lines represent waveforms where the control signal is not turned on, while the continuous blue lines represent signals with the control signal on

In the previous sections, it has been shown that the transmission of acoustic energy through the diode is controlled by establishing, via an RP effect, a water neck between the water layer and the top aluminum disc, filling the air gap and reconfiguring the multilayer system. Our work reveals how this “diode” effect can also be used as a key element of an acoustic switch^41,46,47^, along with a carefully designed phononic crystal (PC) playing the role of band-pass filter (see Fig. 4a).

The principle of an acoustic switch is to control the transmission of a wave (denoted as the signal wave) by another wave (denoted as the control wave)^41,46,47^. The set up in Fig. 4a potentially leads to a wave transistor provided the control wave is of much lower energy than the signal wave, by analogy with an electrical transistor whose gate signal is much weaker than the current flowing between the source and the drain. Here, a switch can be conceived with a control wave used to establish the multilayer reconfiguration via RP (similarly to the excited wave for the above-presented “diode”), in conjunction with a signal wave carrying the information to be transmitted through the device (see the diagrams in Fig. 4a). In order to ultimately transmit just the signal wave, the characteristic frequencies of each wave are chosen to be different, and the PC filter is designed to cut the control wave transmission via the appropriate design of its frequency band gaps. In other words, the band gap frequency range of the PC contains the control wave frequency.

To demonstrate the switch effect, signal waves in the form of wave packets with a carrier frequency *f*_0_ = 35 kHz and with two spectral widths have been chosen. Wide frequency band transducers, centered at 100 kHz and differing from those implemented in the previous section, are used for both the generation and detection of signals. This selected frequency range leads to a PC design with nine alternating layers of Plexiglas and aluminum, with a total thickness of 9 × 30 mm^2^ (see the last section of Supplementary Note 2). The control wave is excited with the same emitting transducer as the signal wave, in the form of a sine function of frequency 95 kHz. Results are presented in Fig. 4b–e, where the emitted and received signals are shown for two modes of operation: (1) when the control wave is on and of sufficiently high amplitude, that is, above the previously discussed diode threshold (blue waveforms); and (2) when the control wave is off (red waveforms), while the signal wave always remains excited. When the control wave is excited, the water bridge turns the diode part into its “passing” state, and the transmission of acoustic energy is allowed. If the control wave is turned off, the water bridge is not created; hence, wave transmission through the diode is inefficient and barely detectable.

In the “on” configuration, both control and signal waves are transmitted through the diode part and are present at the diode–PC interface. The PC then filters out the control wave by means of a Bragg reflection process; only the signal wave can then travel through and be detected by the receiver (see Supplementary Fig. 7 of the Supplementary Note 2). As observed in Fig. 4c, e, only the wave packets and their possible echoes inside the device are transmitted by the switch device, while the control wave is physically filtered out and not present in the received signal.

The switch operation is not instantaneous, that is, similar to the diode effect; this is mainly due to a characteristic time of establishment (or removal) of the water bridge filling the air gap. It is beyond the scope of the present article to model the transient behavior of the diode, although experimental testing has revealed that a fraction of a second is the time typically needed to switch from the passing to the non-passing state. Note, however, that this characteristic time explains why a signal wave of any amplitude cannot establish by itself the water bridge: it is a short wave packet with a characteristic duration of a fraction of milliseconds, shorter than the characteristic time of water bridge formation. Even if the signal wave pulse induces a radiation stress, the application of this stress on the interface is too short to induce a significant motion, due to inertial effects primarily. As such, the signal wave cannot be transmitted by itself, even if it can be of larger amplitude than the control wave.

Although the switch effect is demonstrated and the control wave is generated using a smaller voltage than that of the signal wave (however the average power of the sine control wave is currently higher than that of the signal wave packet), this device is not a fully operational acoustic transistor since the amplification function has not been properly developed. These results on the acoustic switch display the capabilities of such a device in controlling the transmission of one acoustic wave by another acoustic wave. Remarkably, the switch concept proposed here, making use of an acoustic diode based on the RP effect, has the ability to operate over a broad frequency range: the reconfiguration of the multilayer system modifies the impedance jumps that are frequency independent. Note that an acoustical switch based on the RP effect for the migration of a bubble cloud was proposed in ref. ^41^, operating in the frequency range around the bubble resonances. The wide operating bandwidth for the signal wave makes this concept compatible with applications in information processing and with, for example, ultrasonic logic gates.

## Discussion

RP, may be generated by sound or light, has been recently mentioned as an effect to be explored further for its potential in the nonreciprocal transmission of waves^8,13,48^. We have reported on a concept and tested devices associated with an acoustic diode and an acoustic switch, based on the reconfiguration of a multilayer system according to the nonlinear effect of acoustic RP. For the diode effect, we were able to obtain a forward energy transmission close to 1, in keeping the same signal frequency, and a rectification ratio reaching as high as 10^8^ over a wide range of frequencies. The switch is also capable of controlling the transmission of a signal wave packet carrying information. Among the passive devices identified for diode effects, the proposed concept solves nearly all of the existing bottlenecks and allows envisioning actual applications of acoustic diodes, for example, in medical imaging. The tested devices, however, have response times of several hundreds of milliseconds, and moreover they require a specific orientation with gravity. Engineering improvements of the concept to overcome these remaining limitations could consist of downscaling the system to shorten all characteristic times, including response times, in addition to replacing water by a custom-designed nonlinear architected solid with the potential of greatly expanding under application of an ultrasonic field. In addition to paving the way toward tangible applications in acoustics, the reconfiguration concept based on RP and these currently explored improvement scenarios may exert major impacts in other wave fields, such as thermal physics, optics, or optomechanics^48^.

## Supplementary information

Supplementary Information

## Data Availability

The datasets generated during and/or analyzed during the current study are available from the corresponding author on reasonable request.
