# Supplementary material for: Acoustic radiation pressure for nonreciprocal transmission and switch effects
Source: Nat Commun. 2019 Jul 23;10:3292. doi: 10.1038/s41467-019-11305-7 (PMC6650405; doi:10.1038/s41467-019-11305-7)
Supplement: Supplementary file 1 — Supplementary Information [file 41467_2019_11305_MOESM1_ESM.pdf]

Supplementary Information:

Acoustic radiation pressure for nonreciprocal transmission and switch effects

Devaux *et al.*

Supplementary Note 1: Experimental design

Operations of the nonreciprocal device

The experimental set-up consists of a pair of identical piezoelectric transducers, used for the emission and reception of acoustic waves, attached to the multilayer system shown in Fig. 1d in the main article. In this work, three different pairs of flat (non-focused) piezoelectric transducers emitting or detecting longitudinal ultrasonic waves were employed. The results shown in the main text were obtained with two transducers from NDT systems (ref. IDHG029) featuring a piezo-element diameter of 29 mm and a resonance frequency centered at 2.25 MHz. We also conducted tests with two Harisonic (ref. I8-0118-P-SU) transducers (resonance frequency: around 1 MHz; piezo-element diameter: 29 mm). Another pair, composed of two Panametrics transducers (ref. V3052), was also implemented, with a resonance frequency of around 100 kHz and an element diameter of 40 mm. The electrical signal applied to the emitter (piezo-electric transducer) was generated by a Tektronics AFG3022B waveform generator and amplified by a high-power amplifier from Electronics & Innovation E&I (ref.1140LA). For the acquisition process, the signal measured by the receiving transducer was monitored using an oscilloscope (Lecroy LT246ML) connected to a computer via a GPIB link.

Direction- and amplitude-dependent transmission

In the results presented in the main text, the experiment was performed at frequencies of around 2 MHz using the corresponding pair of piezoelectric transducers and the electronic equipment described previously for signal generation and acquisition. Two distinct acoustic frequencies and air-gap thicknesses were tested in order to compare the direction-sensitive and excitation amplitude-dependent transmissions of the nonreciprocal device. For each case, the excitation signal was composed of a sinusoidal signal at the target frequency, amplitude-modulated by means of a triangular signal with a modulation frequency of 40 mHz, leading to peak-to-peak amplitudes varying from 0 to 750 V. This modulation resulted in an amplitude sweep lasting 25 s. The acquisition proceeded by recording the peak-to-peak amplitude of both the emitted and transmitted signals. This operation was carried out for both the denoted "forward" and "backward" directions of transmission by simply inverting

the role (emitter or receiver) of the transducers. As described in the main text, our acoustic diode configuration requests a strong impedance mismatch (or isolation) between the layer 1 and the layer 3. To avoid acoustic energy leakage and preserve the impedance jumps between the layers, but at the same time to realize a device (a multilayer structure) holding itself, five soft rubber pieces with a length of 1 cm and with high damping properties of ultrasonic waves, have been added on the periphery of the air layer as depicted in Fig. 1c,d.

Measurement of the frequency response

The frequency response of the nonreciprocal device has been measured for all three ranges of frequencies presented in this work. For each measurement, a frequency-swept signal is excited by the waveform generator, amplified and sent to the emitter. The range of frequencies is swept within a typical duration of ≈ 20 s, with the exact duration depending on the extent of the frequency range limits. The transmitted signal amplitude and instantaneous signal frequency are quantities measured using the oscilloscope's built-in analysis functions at a rate of 8 points per second. Data are collected by the computer via a GPIB link, and the detected amplitude is then plotted as a function of frequency (see Supplementary Figure 3a or Fig. 3a of the main text).

Operations of the acoustic switch

The acoustic switch presented in the main text (see Fig. 4) has been built by placing a phononic crystal between the top aluminum disc and the receiver of the nonreciprocal device presented in the main text, in considering the "forward" configuration (i.e. receiver on top). We used the pair of identical piezoelectric transducers with a resonance frequency of approx. 100 kHz for the generation and acquisition of acoustic waves. The control and signal waves were emitted by the same transducer, in the respective forms of a continuous sinusoidal signal of frequency $f_c = 95$ kHz and a wave packet with a carrier frequency $f_0 = 35$ kHz and a spectral full-width at half-maximum of 5 and 20 kHz. When both control and signal waves were ON, the signals were summed before their emission. The resulting emitted and transmitted signals visualized on the oscilloscope were collected by the computer via a GPIB link. The excitation amplitude of the control wave was $A_c = 1.1$ V (before amplification),

and the amplitude ratio between the signal and control waves was $A_s/A_c = 4$.

Supplementary Note 2: Additional experimental results

In this section, we provide additional results for the "diode" operation over different frequency ranges than those discussed in the main text, namely in the 100 kHz and 1 MHz ranges. These results show the flexibility of device operations and the universality of the underlying concept. We have also included amplitude-dependent results on the reference case of the device without an air-gap.

Nonreciprocal transmission experiments in the 100-kHz range

A different pair of piezoelectric transducers was introduced to display the universality of the proposed concept and its applicability to various frequency ranges. In this case, the pair of Panametrics V3052 transducers, with a resonance frequency centered at 100 kHz, were used. Note here the extension of the diode effect to an acoustic switch, i.e. combination of the diode effect with a phononic crystal acting as a band-pass filter, has been carried out at the working frequencies of these transducers.

Supplementary Figure 3a illustrates the frequency response of the prototype over the frequency range 60-140 kHz for three air-gap layer thicknesses, $h_a = [2, 3, 4]$ mm. The excitation amplitude equals $V_{in} = 1100$ V. Transmitted amplitudes were generally less than those of the other two frequency ranges under study due to the lower efficiency of both emitter and receiver transducers, as attributed to their broadband characteristic. Nonetheless, the overall behavior of the frequency response resembles that of the other frequency ranges under study. The thinnest air-gap layer, i.e. $h_a = 2$ mm, is associated with the highest values of transmitted amplitudes for a broadband frequency range from 77 to 140 kHz. Increasing the air-gap layer thickness results in smaller transmitted amplitudes, along with the appearance of frequency regions where the water layer is not in contact with the top aluminum disc, hence not allowing the wave to be transmitted (i.e. below 90 kHz or from ≈ 102 to 110 kHz for $h_a = 3$ mm; and below ≈ 95 kHz, from ≈ 102 to 110 kHz, from 118 to 120 kHz, and above 130 kHz for $h_a = 4$ mm).

Let's now make use of the asymmetric ratio in order to estimate the transmission asym-

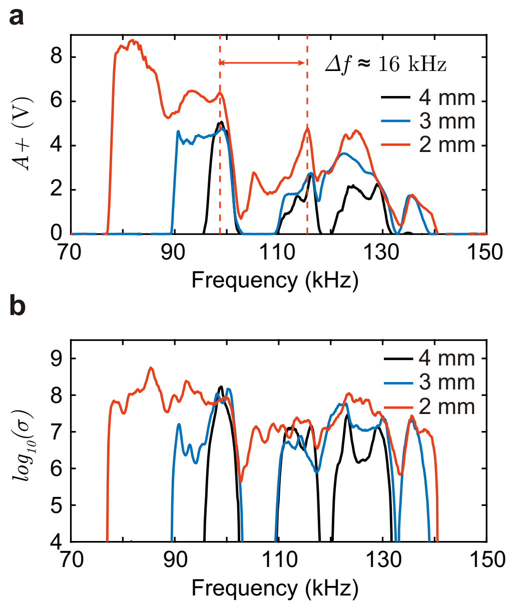

Supplementary Figure 1: Experimental frequency response of the diode effect around 100 kHz. a, Transmitted amplitude in forward direction (A+) as a function of frequency for 3 different air layer heights. The excitation amplitude is $V_{\rm in} = 1100$ V. **b**, Transmission asymmetry ratio $\sigma = A+^2/A-^2$ versus frequency for 3 different air-gap thicknesses.

metry of the prototype built for frequencies of around 100 kHz. This ratio has been defined as: $\sigma = A+^2/A-^2$, where $A+$ and $A-$ are the transmitted amplitudes in the forward and backward directions, respectively. Results on the asymmetric ratio as a function of frequency for three different air-gap thicknesses ($h_{\rm a} = 2, 3, 4$ mm) are shown in Fig.3b. A remarkable asymmetry ratio of $\sigma \approx 10^7$ is observed for all three air-gaps, with a maximum of $\sigma \approx 10^8$. This value typically remains constant as long as the water-layer fills the gap and the contact between water and aluminum is established, although this value is slightly reduced for increasing air-gap thicknesses due to the smaller transmitted amplitude (see the case of $h_{\rm a} = 4$ mm). Note that the results obtained are similar to those for 2 MHz (see Fig.3b in the main article).

Nonreciprocal transmission experiments in the 1-MHz range

The prototype of the main article has also been tested using a pair of piezoelectric transducers with a resonance frequency lying around 1 MHz (Harisonic I8-0118-P-SU). These transducers are flat with a diameter of 29 mm. Their frequency bandwidth is approximately 400 kHz (0.85-1.25 MHz), and their frequency response displays two very close peaks at 0.95 and 1.12 MHz with a small dip in between. This particular feature influences the frequency response of the entire prototype, as shown in the results presented below.

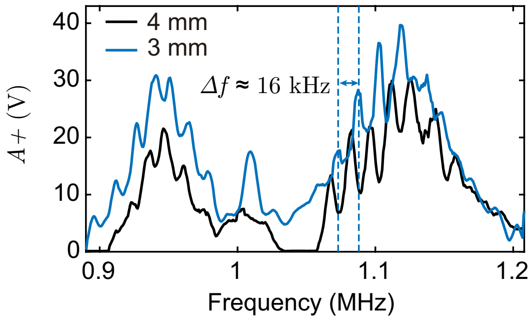

Supplementary Figure 2: Experimental frequency response of the diode effect around 1 MHz. Transmitted amplitude in forward direction (A+) as a function of the excitation frequency for the diode system for 2 different air-gaps thicknesses. The excitation amplitude is $V_{\rm in} = 450$ V.

Supplementary Figure 2 illustrates the transmitted amplitude as a function of frequency using these transducers. The excitation amplitude is $V_{\rm in} = 450$ V and two air-gap thickness values are presented: $h_{\rm a} = 3$ mm and $h_{\rm a} = 4$ mm. The height of the water cavity in this case is $h_{\rm w} = 43$ mm. The oscillations due to Fabry-Pérot resonances of the $h_{\rm w} + h_{\rm a} = 46, 47$ mm water layer are repeated every $\approx$ 16 kHz, as highlighted by the vertical blue dashed lines for the 3-mm air-gap case. For the smallest air-gap, i.e. $h_{\rm a} = 3$ mm, the air-gap layer is completely filled by the water bump for the full range of frequencies, although the transmitted amplitude varies according to the transducer response. As the initial air-gap thickness increases, $h_{\rm a} = 4$ mm, the lateral size of the water bump decreases, thus resulting in slightly lower transmitted values. Moreover, frequencies of around 1.05 MHz have amplitudes lying below the passing threshold, hence no transmission is observed. Similar to the case of 2 MHz, the oscillations in $A+$ are slightly shifted in frequency for the two distinct heights due to the 1-mm difference in effective layer thickness.

Amplitude-dependent transmission in the case without an air-gap

The hysteretic characteristic observed in the transmitted amplitude $A+$ as a function of the excitation amplitude (see Fig.2(c,d) in the main article) is mainly due to the capillary force of water, i.e. the water bump - aluminum contact shows a tendency to be maintained as the excitation amplitude is gradually reduced. Nevertheless, another contribution of hysteresis is observed in the form of a small difference in the detected amplitude $A+$ between the increasing and decreasing excitation amplitude stages. To further illustrate this secondary contribution on the observed hysteresis, the amplitude-dependent transmission results are indicated in Supplementary Figure 1 for two ultrasonic frequencies in a configuration with no initial air-gap between the water layer and the top aluminum layer. The existing hysteresis in the results is attributed to the system temperature change (transducer and water) due to the high-power excitation and associated acoustic energy dissipation. Note that 25 s are required to complete the amplitude sweep, initially increasing from 0 to 750 V and subsequently dropping from 750 to 0 V.

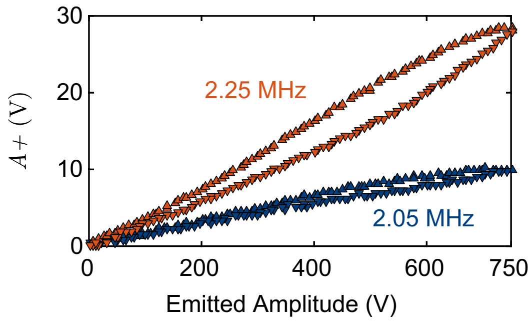

Supplementary Figure 3: Amplitude dependence without air-gap. Transmitted amplitude (A+) in forward direction as a function of the emitted amplitude V_{in} along a cycle of increasing and decreasing amplitudes, for two different excitation frequencies ($f_1 = 2.05$ MHz amd $f_2 = 2.25$MHz), without air-gap.

Additional insight can be gained from a comparison of these results without an air-gap to those presented in Fig.2c in the main article. The maximum transmitted amplitude for a $h_{\text{a}} = 3$ mm air-gap in the forward direction reaches ≈ 26 V at $f = 2.25$ MHz and ≈ 9 V at $f = 2.05$ MHz, which is nearly equal to the maximum transmitted amplitude obtained without an air-gap, ≈ 30 V and ≈ 10 V, thus demonstrating the exceptional transmission efficiency of this device.

Air-water interface deformation height by acoustic radiation pressure: Theory and estimation

The deformation height resulting from acoustic pressure at the center ($r = 0$) of the interface between water and air can be deduced from the acoustic pressure of the incident wave as follows [1] (29):

$$h(r=0) = \frac{A l_{\mathrm{f}}^2 p_{\mathrm{i0}}^2}{8\sigma} \exp\left(\frac{\Delta\rho g}{8\sigma}\right) E_1\left(\frac{\Delta\rho g}{8\sigma}\right), \tag{1}$$

where σ is the water surface tension, g is the gravity constant, $\Delta\rho \approx \rho_{\mathrm{w}}$ is the differential density between water and air, E_1 the integral exponential function, l_{f} the characteristic length of the incident beam and p_{i0} is the incident pressure amplitude on the surface. A denotes the factor of acoustic radiation force ($\Pi = 2Ap_{\mathrm{i0}}^2$),

$$A = \frac{1}{\rho_{\mathrm{w}} c_{\mathrm{w}}^2} \frac{\rho_{\mathrm{w}}^2 c_{\mathrm{w}}^2 + \rho_{\mathrm{a}}^2 c_{\mathrm{a}}^2 - 2\rho_{\mathrm{w}}\rho_{\mathrm{a}} c_{\mathrm{w}}^2}{(\rho_{\mathrm{w}} c_{\mathrm{w}} + \rho_{\mathrm{a}} c_{\mathrm{a}})^2}. \tag{2}$$

where the sound speed and density for water and air are, c_{w}, c_{a} and ρ_{w}, ρ_{a}, respectively. These formulae have been quantitatively tested in [1].

An initial measurement was conducted to determine the acoustic pressure of an acoustic wave at an air-water interface. An experimental system, composed of a piezoelectric transducer in the 100-kHz range (Panametrics V3052) attached to a multilayer system, was used for this purpose. The multilayer was formed by two successive layers, i.e. a 3-mm thick aluminum layer on the bottom part and a water cavity of $h_{\mathrm{w}} = 43$ mm above. A Polytec OFV505 laser vibrometer with a Polytec OFV-5000 controller was deployed. The laser vibrometer was positioned perpendicular to the interface and a small reflective film was placed on the water surface, as shown in the schematic diagram of the experimental set-up in Supplementary Figure 4.

In order to determine acoustic pressure from the acoustic velocity output by the vibrometer on the water surface, we assumed a total reflection of the wave at the air-water interface, hence the incident and reflected acoustic pressures are identical: $p_{\mathrm{i0}} = p_{\mathrm{r0}}$. Similarly, the measured total acoustic velocity is assumed to equal the contribution of the incident and reflected particle velocities: $v_{\mathrm{m}} = v_{\mathrm{i}} + v_{\mathrm{r}}$. Lastly, in considering the relation between acoustic pressure and velocity, the acoustic incident pressure is determined using the relation: $p_{\mathrm{i0}} = \rho_{\mathrm{w}} c_{\mathrm{w}} v_{\mathrm{m}}/2$. Note that the excitation amplitude ($V_{\mathrm{in}} = 70$ V) was small enough to

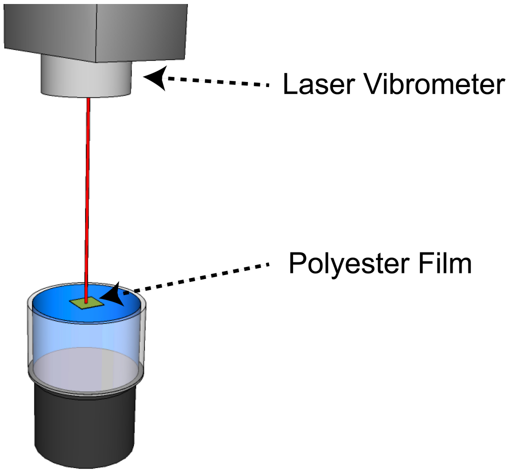

Supplementary Figure 4: Experimental setup: Acoustic velocity measurement. Schematic of the experimental setup to determine the acoustic velocity at the air/water interface. A polyester film is placed on the interface to enhance reflectivity of the laser emitted by the vibrometer.

avoid lateral displacement of the reflective film caused by deformation at the interface due to radiation pressure. Supplementary Figure 5 shows the measured particle velocity at the air-water interface as a function of frequency. Resonant peaks every kHz due to Fabry-Pérot resonances are clearly visible in these results, as indicated by vertical dashed lines. This result makes it possible to measure the acoustic incident pressure and moreover is used as a model input (Eq. 1) to estimate the expected deformation height at higher amplitudes.

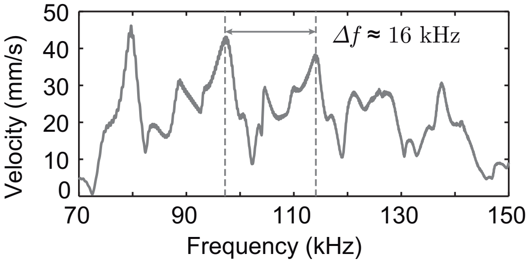

Supplementary Figure 5: Acoustic velocity at the air/water interface. Measured acoustic velocity as a function of frequency for a small excitation amplitude, $V_{\text{in}} = 70$ V. Dashed vertical lines highlight the frequency spacing between consecutive peaks originated by Fabry-Pérot resonances.

The estimated deformation height for an air-water interface at frequencies of around

100 kHz has been calculated using Eq. 1. All parameters entering into the calculations are set as follows: density and speed of sound in air and water $\rho_a = 1.2$ kg/m^3, $\rho_w = 1000$ kg/m^3, $c_a = 340$ m/s, $c_w = 1500$ m/s; surface tension between water and air at 20°C $\sigma = 0.072$ N/m; gravity constant $g = 9.81$ m.s^{-2}; and characteristic width of the beam $l_f = 1.47\lambda$, with λ being the wavelength in water. In addition, the value of the incident acoustic pressure has been calculated from the acoustic velocity measurement presented in Supplementary Figure 5 and then linearly scaled for an excitation amplitude of $V_{\text{in}} = 1100$ V. The results obtained are shown in Supplementary Figure 6. Comparing this prediction

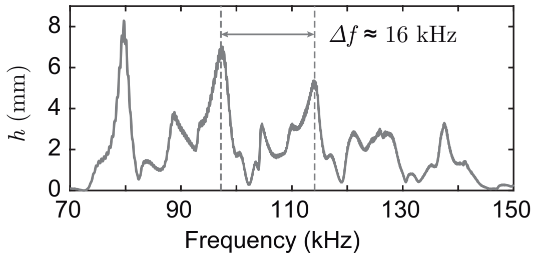

Supplementary Figure 6: Estimated height from the model reported in [1]. Deformation height, h, of the water/air interface as a function of the frequency of excitation for an input amplitude $V_{\text{in}} = 1100$ V.

to the experimental transmission results from Supplementary Figure 3a leads to a good agreement, especially considering the frequency values of the resonant peaks due to Fabry-Pérot resonances and the deformation height relative to the initial air-gap thickness. The fact that the interface is strongly deformed at large pressure amplitudes leads to a disturbance of the regularly-spaced (high quality factor) resonance characteristic and ultimately to a deviation from model behavior. At an excitation amplitude of 70V, a peak incoming acoustic pressure of 30 kPa can be estimated from the 40 mm/s peak velocity, corresponding to a power of 600 W/m2. If we linearly extrapolate these estimates, we reach for instance a peak pressure of 300 kPa and a power of 0.6 W/cm2 at a 700V excitation amplitude. This value is comparable to the cavitation threshold of 0.5 W/cm2 at 20 kHz, but this threshold increases with frequency, so it is expected higher at 100 kHz. We did not observe cavitation effects in our experiments.

Design of the acoustic switch and characterization of the phononic crystal

Let's now turn our focus to the phononic crystal, which was designed to act as a bandpass filter in the acoustic switch presented in the main article. Let's recall the working principle of this switch for a better understanding of both the role and design of the phononic crystal: the transmission of an acoustic wave carrying particular information, denoted the signal wave, is controlled by another wave, called the control wave, which is responsible for reconfiguring the multilayer system via the radiation pressure effect. These two signals lie at different frequencies. Since the control wave is only necessary to generate the water neck and allow for signal wave transmission, the main goal of the phononic crystal design is twofold, i.e. preventing propagation of the control wave through it, and allowing the signal wave to be transmitted. With this in mind, the signal wave, which may have a broadband frequency range, consists of a wave packet containing frequencies belonging to the first propagative band of the phononic crystal (f_0 = 35 kHz), while the control wave, which will be a continuous sinusoidal signal (f_0 = 95 kHz), has a frequency within the band-gap of the phononic crystal. The phononic crystal is formed by nine alternating cylindrical layers of PMMA and 30-mm thick aluminum (l_p = 10 mm, l_a = 20 mm), with a diameter d = 30 mm. The frequency response of the phononic crystal can then be calculated numerically using the Finite Element Method (FEM) and experimentally by applying a swept sinusoidal signal ranging from 10 to 150 kHz. These results are shown in Supplementary Figure 7 and reveal good agreement between numerical calculations and experimental output. The first propagative band extends to 60 kHz, while the band-gap is located from 60 to 120 kHz.

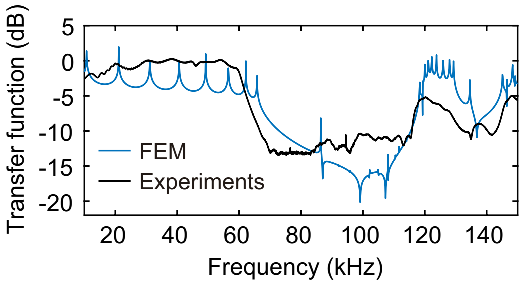

Supplementary Figure 7: Phononic Crystal frequency response. Frequency response of the PC comparing numerical simulations (FEM) and the experimental results (EXP).

Supplementary References

[1] Issenmann, B. Wunenburger, R. Manneville, S. & Delville, J. P. Bistability of a compliant cavity induced by acoustic radiation pressure. *Phys. Rev. Lett.* **97**, 074502-4 (2006).
